# Attitude, reporting behavour and management practice of occupational needle stick and sharps injuries among hospital healthcare workers in Bale zone, Southeast Ethiopia: a cross-sectional study

**DOI:** 10.1186/s12995-015-0085-2

**Published:** 2015-12-03

**Authors:** Tolesa Bekele, Alem Gebremariam, Muhammedawel Kaso, Kemal Ahmed

**Affiliations:** Department of Public Health, College of Medicine and Health Sciences, Madda Walabu University, Oromia, Ethiopia; Department of Public Health, College of Medicine and Health Sciences, Adigrat University, Adigrat, Tigray Ethiopia

**Keywords:** Reporting behavior, Injury, Healthcare workers, Bale zone, Oromia, Ethiopia

## Abstract

**Background:**

Although the prevalence of blood borne pathogens in many developing countries is high, documentation of infections due to occupational exposure is limited. Seventy percent of the world’s HIV infected population lives in Sub-Saharan Africa, but only 4 % of cases are reported from this region. Under reporting of needle stick and/or sharps injuries in healthcare facilities was common.

**Methods:**

An institutional based cross-sectional study was conducted in December 2014 among healthcare workers in four hospitals of Bale zone, Southeast Ethiopia. A total of 362 healthcare workers were selected randomly from each of the working departments. Data were collected using self-administered questionnaire and were entered using Epi-Info version 3.5 and analysed using SPSS version 20.0. Multivariable logistic regression analysis was used to identify independent effect of each variable on the reporting behaviour of needle stick and/or sharp injury.

**Results:**

Nearly six out of ten injuries (58.7 %) were not reported to the concerned body. The main reasons for not reporting the injuries were time constraint (35.1 %), sharps which caused injury were not used on any patient (27.0 %), the source patients did not have disease of concern (20.3 %), and lack of knowledge that it should be reported (14.9 %). Half of healthcare workers (HCWs) those who experienced injury had sought medical care next to self based action. Respondents with monthly salary of 450 to 1000 Ethiopian Birr (1 US Dollar = 22.00 Ethiopian Birr) were about six times more likely to report occupational needle stick and/or sharps injury (NSSI) than HCWs with salary of 2001 to 8379 birr (AOR = 5.73). However, HCWs who had no knowledge about probability of infection transmission through NSSI and not taking any self based measures after occurrence of injury were 45 % (AOR = 0.55) and 93 % (AOR = 0.07) less likely to report occupational injury than their counterparts, respectively.

**Conclusions:**

Occupational needle stick and/or sharps injuries are common among HCWs at the study area. Even though majority of respondents were concerned about the risk of NSSI exposure, most respondents did not report it to the concerned body. Therefore, provision of on job training on the risk of occupational NSSI exposure may strengthen HCWs to practice timely reporting and its management in case of occupational injury exposure.

## Background

World Health Organization (WHO) in 2007 endorsed the Global Plan of Action on Workers’ Health from 2008 to 2017 to provide political framework for development of policies, infrastructure, technologies and partnerships for achieving basic level of health protection in all workplaces throughout the world. The Global Plan of Action addresses all aspects of workers’ health, including primary prevention of occupational hazards, protection and promotion of health at work, employment conditions and improving the response of health systems to workers’ health. In such a way it links occupational health to public health [1].

While as many as twenty blood borne pathogens can be transmitted through accidental needle and/or sharps injury, the potentially life threatening are Human Immunodeficiency Virus (HIV), hepatitis B virus (HBV) and hepatitis C virus (HCV) [2]. Needle stick and sharps injury (NSSI) is an injury with needles, scalpel blade, catheter stylet or other pointed objects which were used for any invasive procedure with a patient and contaminated with blood or body fluids. Sharps injuries are hidden problem; after it happens most of workers forget and get on with their work. Neither the magnitude of the risk of needle stick and sharps injury nor the practices associated with it has been well understood among HCWs [3].

A number of policy strategies are available for avoiding disease burden associated with NSSI, including vaccination against HBV, post exposure prophylaxes (PEP) for HBV, HCV and HIV, reducing the number of injections and invasive procedures where appropriate, using safer devices and properly disposing of needles and other sharps. Despite the presence of many opportunities to reduce the disease burden, the full impact of NSSI is not yet recognized [4]. Most of people at risk for occupational exposures are in developing countries where there is paucity of reporting standard protocols. In addition, poor knowledge and practices related to the risk and hazard of sharps injuries substantially contribute to the probability of the injuries [5].

Although the prevalence of blood borne pathogens in many developing countries is high, documentation of infections caused by occupational exposure in these countries is scarce. Seventy percent of the world’s HIV infected population lives in Sub-Saharan Africa but only 4 % of worldwide cases of occupational HIV infection are reported from this region [6]. Underreporting of sharps injuries by employees is documented in different literatures. The magnitude of underreporting ranges from 22 to 99 % [7]. There are many reasons why healthcare workers do not report sharp injuries. They may perceive that the injuries or the source patients are low risk, they may fear the diseases to which they have potentially been exposed, they may have concerns about job security or the extra paperwork and time involved in follow-up. In addition, they may lack information and training about appropriate reporting procedures or the reporting procedures themselves may be inadequate [8, 9].

Ethiopian healthcare facilities do not have adequate reporting system. This is why most of incidents are not reported and inadequately treated. Tangible evidence on the reporting system and its management in healthcare facilities is very limited and varies from setting to setting. There are few studies conducted on occupational NSSI exposure. However, the reason why HCWs do not report incidents of sharp injuries was not yet well addressed. To the best of our knowledge, there is no any available data reported before about occupational NSSI reporting behaviour and its management among HCWs from the study area.

## Methods

The study was carried out in December 2014 at four hospitals namely Ginnir, Robe, Delo mena and Goba. The four hospitals are found in Bale Zone, Oromia Regional State, at Southeast of Ethiopia. By the year 2014, these four hospitals were offering different types of healthcare services for the surrounding community. Hospital based cross-sectional study design was employed. All healthcare workers working in the four hospitals who have direct contact with patients or equipments used on patients were involved in the study. These were doctors, anaesthetists, health officers, nurses, midwives, laboratory personnel, laundry workers and waste handlers. All individuals out of work during data collection due to annual leave and those who could not respond to the questions due to illness were excluded from the study.

The sample size was determined using single population proportion formula with the following assumptions: the proportion of HCWs who experienced needles stick and/or sharp injury in the previous 12 months was 31.0 % [10], 1.96 values of 95 % confidence interval, 5 % margin of error and 10 % non-response rate. The total sample size became 362.

All hospitals in Bale zone were included in the study. Before selection of study participants, first we obtained the list of workers and categorized them into their specific working department. Then proportionate allocation to size was used for each department to share the total sample size. Study participants who fulfil the inclusion criteria were selected by simple random sampling technique using the list of workers taken from each working department.

Questionnaire was developed by referring published literatures [10–13]. The questionnaire was first written in English and then translated to Afan Oromo and Amharic (local languages) by experts in both languages and then retranslated to English to check for consistency. Eight data collection facilitators (BSc nurses) were assigned to the four hospitals (two per hospital). Pre-testing was done on 5 % of the same source population other than the sampled population in Goba and Robe hospitals. Based on the pre-test result, necessary correction was made for the questionnaire. Data were collected using self-administered questionnaire.

The questionnaire included background information, attitude about risk of NSSI, its management and reporting behaviour of the respondents. Finally, the questionnaire was administered by data collection facilitators who were trained for 1 day ahead of data collection. Supervision during data collection was done by investigators to see how the data collection facilitators were handling data collection process. Each filled questionnaire was checked for its completeness and consistency on daily basis.

The collected data were checked for completeness and consistency by the principal investigators before data entry for analysis. Prior to data entry, each questionnaire was given a unique code by principal investigator. The principal investigator prepared template and entered data using Epi Info version 3.5 and 20 % of the data were double entered in computer software. Data cleaning were done to check missing values and inconsistencies by running frequencies for each variable. Cleaned data were exported from Epi Info to Statistical Program for Social Sciences (SPSS) version 20.0 for analysis. Frequency distribution and percentage were used to present the results of uni-variate analysis. Logistic regression model was used to identify the independent effect of each predictor variable on the dependent variable. Those variables which were significant on bi-variate analysis (P-value < 0.05) were entered to multivariable logistic regression analysis. The association between the reporting behaviour of NSSI and independent variables was determined using odds ratio (OR) with 95 % confidence interval (CI). The level of significance was taken at α = 0.05.

Ethical clearance was obtained from the Ethical and Review Committee of Madda Walabu University, College of Medicine and Health Sciences. An official permission was communicated through formal letter from Madda Walabu University Research and Community Service Directorate Office to the respective hospital. Written consent was secured from each participant. Participants were told that the information provided was confidential and that their identities were not revealed in association with the information they provided.

## Results

### Socio-demographic characteristics of participants

From a total of 362 HCWs working in four hospitals, 340 fully responded the questionnaire, giving a response rate of 93.9 %. The mean age of the respondents were 28.3 (SD = ± 7.7) years. More than eight out of ten (82.1 %) of the respondents were found in the age group of 18 to 32 years. Majority of the participants were Orthodox Christian (62.1 %) by religion and were Oromo (79.1 %) by Ethnicity. Nearly half of the respondents (49.7 %) were nurses by profession (Table 1).

**Table 1:** Socio-demographic characteristics of hospital healthcare workers in Bale zone, December, 2014 (*n* = 340)

Socio- demographic characteristics	Frequency (n)	Percentage (%)
Participants’ working hospital		
Ginir	90	26.5
Goba	115	33.8
Delo mena	70	20.6
Bale Robe	65	19.1
Sex of respondents		
Male	153	45.0
Female	187	55.0
Age group of participants		
18–32	279	82.1
33–47	44	12.9
48–62	17	5.0
Marital status		
Ever married	191	56.2
Never married	149	43.8
Religion		
Orthodox	211	62.1
Muslim	67	19.7
Protestant	58	17.0
Wakefata	4	1.2
Ethnicity		
Oromo	269	79.1
Amhara	59	17.3
Gurage	6	1.8
Tigrie	3	0.9
Others (Harari, Somali and Wolayita)	3	0.9
Educational level		
Grade(5–12th)	55	16.2
College diploma and above	285	83.8
Monthly salary		
450–1000	77	22.7
1001–2000	60	17.6
2001–8379	203	59.7

### Attitude about risk of needle stick and sharp injury

Almost all of the respondents 334 (98.2 %) knew about the risk of NSSI exposure. Among the HCWs who knew about the risk, 273 (81.3 %), 46 (13.7 %) and 17 (5.1 %) rated as high, moderate and low risk, respectively. More than eight out of ten 283 (83.2 %) respondents perceived that NSSI is avoidable. The main perceived prevention methods of NSSI were proper disposal of the used needles (35.7 %), careful collection of needles and/or sharps (25.1 %) and adherence to universal precautions (21.2 %). Significant proportion (16.2 %) of respondents also perceived that personal protective equipment and other methods such as not recapping needle and sterilizing before disposal (1.8 %) as a method of prevention. Nearly all respondents 338 (99.4 %) knew that diseases can be transmitted through NSSI exposure. Respondents were asked about type of diseases which can be transmitted due to occupational NSSI exposure. Almost eight out of ten (81.1 %) HCWs reported at least three diseases including HIV, HBV and HCV. Only ten respondents reported that malaria can be transmitted through occupational NSSI.

Respondents were asked about what contributed to NSSI in their respective hospital. Thirty one percent of the participants reported that lack of personal protective equipment in the working place could put HCWs at increased risk of injury. Similarly, excess workload (28.5 %), improper disposing of needles into safety box after use (22.9 %), and other such as over crowed in working department, waste handling and lack of hand washing facility in the working unit account for 17.6 % of injuries. Most of the respondents (95.6 %) reported that safety box was available in their working department. Whereas 148 (43.5 %) of the respondents reported that there was no occupational NSSI reporting protocol in their working department. Only 20 % of HCWs received their vaccination after starting their career at the hospital. From a total of the respondents, 119 (35.0 %) got tested for HIV, HBV and HCV at the time of the study. Of those who got tested, nearly half (54.9 %) of the respondents had been tested for at least one of the three diseases (HIV, HBV and HCV).

### NSSI management and reporting behaviour of the respondents

Of those HCWs experienced NSSI, majority 107 (84.9 %) of them took self based measures. The most common self based actions taken were washing the injured body part with soap and water (53.3 %), washed with iodine or alcohol solution (42.1 %), and HIV testing (40.2 %). However, 19 (15.1 %) of HCWs did not take any action for their injury. Nearly six out of ten (58.7 %) respondents did not report the accident of their injury to concerned body. The main reasons for not reporting the injury were: time constraints (35.1 %), sharps caused injury were not used on any patient (27.0 %), it was used on patient but the patient did not have disease of concern (20.3 %) and lack of knowledge that it should be reported (14.9 %).

Half of the HCWs who experienced injury had sought medical care next to self based action. Of those who sought medical care, 39.7 % took post exposure prophylaxes such as antiretroviral agents (zidovudine) and tetanus anti-toxoid (TAT). Among the NSSI events reported to infection prevention department, 94.2 % were reported immediately after the injury (Table 2).

**Table 2:** Management and reporting behaviour of NSSI among hospital healthcare workers, Bale zone, December, 2014

Variables	Frequency (n)	Percentage (%)
Ever experienced NSSI at work (*n* = 340)		
Yes	126	37.1
No	214	62.9
Self management taken after injury (*n* = 126)		
Yes	107	84.9
No	19	15.1
Type of action taken after injury^a^		
Washed with soap and water	57	53.3
Washed with iodine or alcohol	45	42.1
Get tested for HIV	43	40.2
Take post exposure prophylaxis (PEP)	25	23.4
Take tetanus anti-toxoid (TAT)	31	29.0
Squeezing to extract more blood	10	8.5
Applying pressure to stop bleeding	18	16.8
Ever reported NSSI to concerned body (*n* = 126)		
Yes	52	41.3
No	74	58.7
Time of injury reported (*n* = 52)		
Immediately after injury	49	94.2
Late before going off work place	2	3.8
After two days of injury	1	1.9
Reasons for not reported injury^a^		
Being too busy at the time of injury	26	35.1
Sharps caused injury never used on patient	20	27.0
Used on patient but was not disease of concern	15	20.3
They did not know as they should report	11	14.9
They did not know how to report	7	9.5
Their colleagues told them not to worry	1	1.4
Sought medical care after injury (*n* = 126)		
Yes	63	50.0
No	63	50.0
Treatment given (*n* = 63)		
Tested for different diseases	15	23.8
Pre-test counselling	4	6.3
Post-test counselling	19	30.2
PEP was given and follow-up started	25	39.7

^a^Each of the percentages does not add up to 100 % because respondents could choose several responses which could be more than one

### Factors associated with reporting behaviour of needle stick and/or sharp injury

On bi-variate analysis respondents from Goba hospital were four times more likely to report occupational NSSI compared to respondents from other hospitals in the study area. But this becomes not significant after controlling other variables in the multivariable analysis (Table 3). Respondents with educational status (5–12th grade), presence of occupational injury reporting protocol and safety guideline in working department were the predictors of reporting the NSSI events compared to their counterparts. But these were also not significant on multivariable analysis. Respondents with monthly salary of 450 to 1000 Ethiopian Birr (1 US Dollar = 22.00 Ethiopian Birr) were about six times more likely to report occupational NSSI than HCWs with salary of 2001 to 8379 birr (AOR = 5.73, 95 % CI: 1.71, 19.23). However, HCWs who did not know that diseases can be transmitted through needle stick and/or sharp injury and who did not take self based measures after occurrence of injury were 45 and 93 % less likely to report occupational injury than their counterparts (AOR = 0.55, 95 % CI: 0.47, 0.68) and (AOR = 0.07, 95 % CI: 0.01, 0.63), respectively (Table 3).

**Table 3:** Multivariable analyses of factors associated with NSSI reporting behaviour among hospital healthcare workers in Bale zone, December, 2014

Variables	Reporting of NSSIs exposure	
	Crude OR (95 % CI)	Adjusted OR (95 % CI)
Working hospital		
Ginir	1.63 (0.52, 510)	1.04 (0.27, 4.05)
Goba	4.1 (1.37, 12.30)^*^	2.08 (0.46, 9.44)
Delo mena	1.62 (0.44, 5.95)	0.48 (0.08, 2.90)
Bale Robe	Ref	Ref
Educational level		
Grade(5-12th)	2.95 (1.07, 8.11)^*^	7.54 (0.46, 12.56)
College diploma and above	Ref	Ref
Monthly income		
450–1000	3.05 (1.17,7.98)^*^	5.73 (1.71, 19.23)^*^
1001–2000	1.68 (0.68, 4.15)	2.67 (0.88, 8.10)
2001–8379	Ref	Ref
Diseases can be transmitted by NSSI		
Yes	Ref	Ref
Do not know	0.70 (0.31, 0.89)^*^	0.55 (0.47, 0.68)^*^
Self management taken after injury		
Yes	Ref	Ref
No	0.06 (0.01, 0.47)^*^	0.07 (0.01,0.63)^*^
Presence of protocol for reporting		
Yes	2.62 (1.26, 5.46)^*^	2.17 (0.74, 6.34)
No	Ref	Ref
Safety guidelines available		
Yes	2.87 (1.34, 6.15)^*^	2.39 (0.95, 6.07)
No	Ref	Ref

^*^Significant at *P* < 0.05, *Ref* Reference

## Discussion

Nearly four out of five respondents in this study rated the level of risk of infection after exposure to NSSI is as high risk. This result is much higher than the study done in Royal College of nursing in London in which 15 % of the respondents perceived that the level of risk of contracting a blood borne that disease from their last injury is as high risk [14]. Almost all respondents (99.4 %) knew diseases can be transmitted through NSSI. This finding is comparable with the study done in Felege Hiwot Referral hospital in Bahir Dar (98.5 %) [10]. It is also indicated in other study that most of HCWs know the possibility that NSSI could lead to the contracting of diseases such as HIV, hepatitis B and C [15].

The current study found that two fifth of the respondents reported that there was no reporting protocol for NSSI in their respective working department. In similar way the study done in two hospitals of Tanzania reported that more than half of the observed hospital departments did not have guidelines for prevention and management of occupational exposure to HIV infections [16]. Lack of reporting protocol supplies may seriously hampers reporting effort and puts both patients and HCWs at increased risk of infection. Current study showed that there was low HBV vaccination coverage (20.3 %) in the study area. This result is much lower compared to study done in Pakistan where 86.3 % of HCWs received their vaccination after starting their job at the hospital [17]. It is recommended that the need for immunization against HBV in the start of career in healthcare settings [4] but no such policy is implemented in Ethiopian healthcare facilities and specifically at the study area.

In this study it was revealed that half of the injured workers did not seek medical care after they sustained NSSI. This is inconsistent with the study done in Mongolian public hospitals in which out of the respondents who reported at least one NSSI in the previous 3 months, 35.9 % answered as they did not seek medical assistance after the occurrence of an injury [12]. This may imply that a system should be introduced in healthcare facilities to ensure that all healthcare workers should be pre-informed where to seek medical treatment after the occurrence of NSSI.

Nearly 60 % of the respondents did not report the injury to the concerned body. The main reasons stated for not reporting were time constraint, needles or sharps caused injury were not used on patients and low perceived risk of diseases transmission due to injury. This result is almost in agreement with Ahmadabad (64 %) [18], and in Alexandria (74.7 %) [19]. But it is higher as compared to the report from Felege Hiwot Referral hospital in Bahir Dar, Ethiopia (46.1 %). The reason for not reporting the occurrence of injury was; the victims perceived that the patient had low probability of being HIV positive especially when the disease status of the patient is unknown [10]. Barriers that hinder reporting of injuries should be appropriately identified in order to provide counselling and treatment for HCWs after exposure to the event.

Respondents who had lower monthly salary were six times more likely to report occupational NSSI. The possible justification might be those HCWs with lower income may seek help from employer to get medical expenses. In addition, those workers with high income may not report to employer to seek money for treatment since they can afford expenses by themselves. Healthcare workers who did not know probability of diseases transmission through NSSI had 45 % lower odds of reporting their injury. Similarly, those who did not take self based measures after occurrence of injury were 93 % less likely to report occupational injury to the concerned body. This may suggest that providing on job training for HCWs on the importance of reporting after injury could improve the victim’s medical seeking behavior.

In interpreting the findings of this study, taking the limitations into consideration is important. Since the study was based on self-reported data in assessing reporting behaviour and management of occupational NSSI exposure; a common threat to the validity of the self-report that can lead to information bias is social desirability and recall bias. In addition, cross-sectional study by its nature cannot establish temporal cause and effect relationships.

## Conclusions

Based on the findings of the study it is concluded that HCWs working in the four hospitals of Bale zone are at increased risk of blood borne pathogens infection due to NSSI. Knowledge of the risk of blood borne pathogens transmission due to occupational exposure and whom to contact in the event of NSSI exposure seems to affect practices of HCWs in relation to its management and timely reporting. Therefore, more rigorous control measures and occupational injury prevention and its management programs need to be implemented by national and institutions. In addition, monitoring and control of health of healthcare workers in order to promote decent and safe work throughout the health facilities is important. Furthermore, regular reporting, provision of hepatitis B immunoglobulin (HBIG) for victims and evaluation of occupational NSSI exposures need to be introduced in the study area.
